# Challenges in implementation of public policies in aging and dementia in Peru

**DOI:** 10.1371/journal.pgph.0002345

**Published:** 2023-09-07

**Authors:** Monica M. Diaz, Maritza Pintado-Caipa, Patricia J. Garcia

**Affiliations:** 1 Department of Neurology, University of North Carolina at Chapel Hill School of Medicine, Chapel Hill, North Carolina, United States of America; 2 Global Brain Health Institute, University of California, San Francisco, San Francisco, California, United States of America; 3 School of Public Health, Cayetano Heredia University, Lima, Peru; PLOS: Public Library of Science, UNITED STATES

Due to population aging and health transitions, people with dementia in Latin America and Caribbean region (LAC) are expected to increase from 7.8 million in 2013 to over 27 million by 2050 [1]. Fragmented healthcare systems in the LAC region make it challenging to provide adequate dementia care. However, implementing preventive interventions to reduce the burden of dementia can help strengthen services for aging populations [2]. It is crucial to establish public policies that allocate governmental resources to implement these strategies.

Dementia is a common condition that occurs in older age presenting as memory impairment with behavioral and psychological symptoms leading to dependence on caregivers and presents significant medical, social, and economic challenges worldwide [2]. The Lancet Commission’s 2020 report “Dementia prevention, intervention, and care” identified twelve modifiable risk factors for dementia, which can serve as targets for Ministries of Health. By addressing these factors and focusing on high-risk groups, it is possible to prevent or delay up to 40% of dementias worldwide [3]. However, if countries are to attain such progress in ensuring those with dementia receive these essential services, they must enact public health programs and support regional dementia research networks [4].

The World Health Organization, the Pan American Health Organization, and Alzheimer’s Disease International have recommended that countries devise their own national policies and strategies to mitigate the impact of dementia. Costa Rica took the lead in the LAC region by establishing a National Plan for Alzheimer’s Disease and Related Diseases in 2014 [5], and by implementing community and clinic memory centers, training for caregivers and by giving access to medications for Alzheimer’s disease through the social security system [6]. Argentina launched the National Strategic Plan for a Healthy Brain, Alzheimer’s Disease and other Dementias in 2016, focusing on improving the training of professionals, caregivers and family members, as well as enhancing access to diagnosis and treatment [7]. Chile implemented a National Plan for Dementia in 2015 [8], which has improved strategies for dementia prevention, diagnosis, palliative care and the training of healthcare professionals. Peru, along with other LAC countries, has lagged behind in the implementation of such policies and strategies.

Peru, like many LAC countries, is experiencing rapid aging with over 33 million inhabitants and with 4.3 million people aged 60 or older. This number is expected to double by 2050; in a recent National House Survey conducted by the Peruvian Office of Statistics and Informatics, nearly 40% of households reported at least one older adult over age 60, with these older adults often being the head of household and the main source of income in 25% of all households [9]. Over a 25-year period, disability-adjusted life years due to Alzheimer’s disease in Peru increased by 157% [10], consistent with reports from other LAC countries [6]. Contributing factors to this increase may include unstable health and social systems and higher health risk indicators in Peru.

The burden of dementia in Peru places significant strain on caregivers, mostly families without prior caregiving training, who are primarily responsible for caring for individuals with dementia, often without receiving any payment. Only 6% of the population of Peru currently have access to private health insurance. Homes or skilled nursing facilities for older adults funded by the public health sector are uncommon (only 8 were reported in 2020) and many skilled nursing facilities are only accessible to individuals with private health insurance (322 private facilities as of 2020), likely due to inadequate governmental financial resources [11]. In 2018, only 277 geriatricians, specialists in caring for older adults, existed to serve the nearly 4 million older adults of Peru [12]. Moreover, dementia incurs a high economic burden in Peru. A study conducted in a private clinic in Lima, Peru, revealed that the monthly cost of caring for patients with dementia was 2.5 times higher than the minimum wage in 2015 [13]. Thus, policy reform is urgently needed in Peru to address the nationwide impact of dementia.

**Fig 1:**
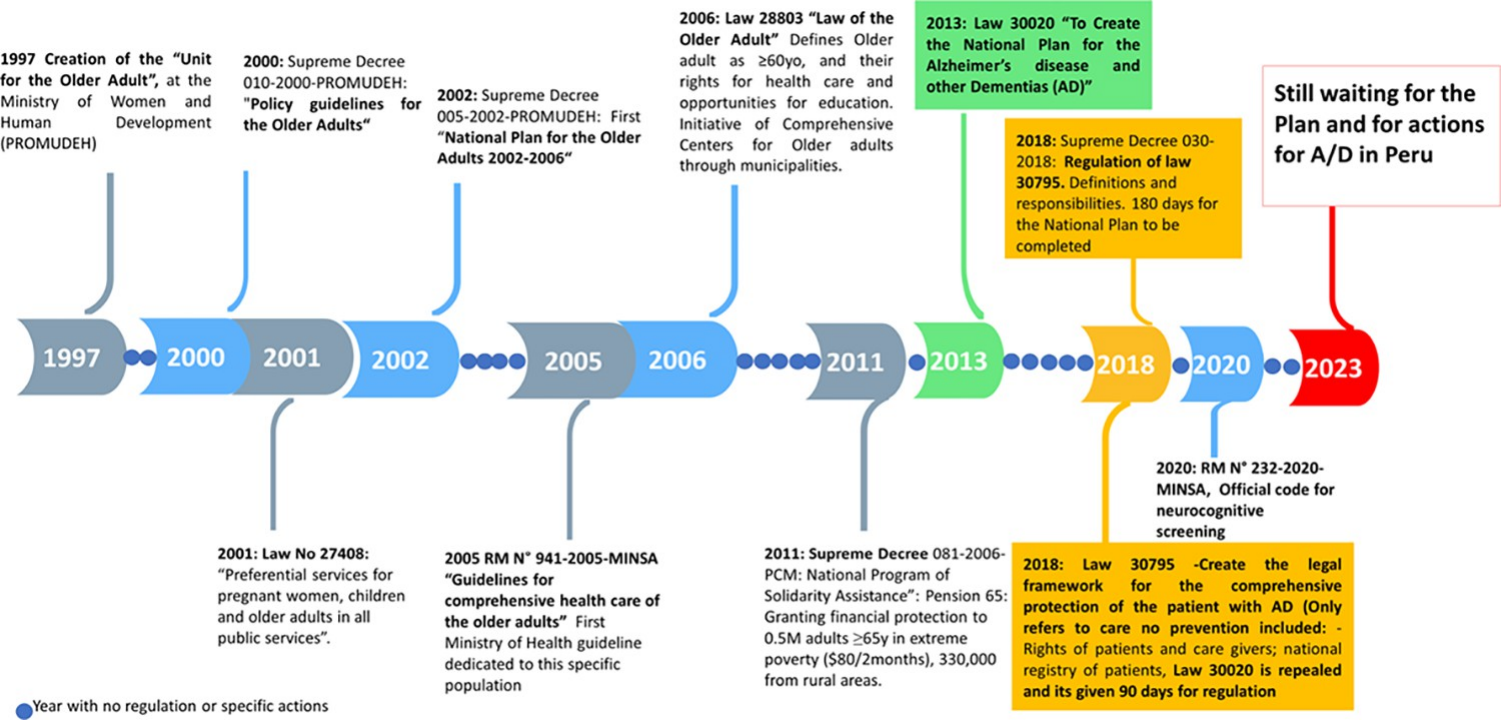
Timetable of policies related to older adults and dementias in Peru.

In Peru, initial policies geared towards the aging population date from 1997 (Fig 1). In 2000, the "Policy Guidelines for the Older Adult" emphasized the need to design and implement multisectoral promotion and prevention programs to improve strategies for healthy aging. Unfortunately, these guidelines were not backed with the appropriate budget and were not implemented. In 2011, the Peruvian Prime Minister’s office released the National Program of Solidarity Assistance: Pension 65, which allocated funding to provide financial assistance to 500,000 adults over the age of 65 living in extreme poverty, including 330,000 older adults in rural areas. This program is still active, and evaluations indicate that at least one-third of the economic support is spent on food [14].

In 2005, the Ministry of Health (MINSA) released the “Guidelines for Comprehensive Healthcare for Older Adults”, which briefly mentioned cognitive impairment. It was not until 2013 that Congress passed a law (30020) to establish a “National Plan for Alzheimer’s Disease and other Dementias (A/D)”. This law included essential measures addressing dementia prevention, management, healthcare provider training, community awareness, and dementia research in Peru. However, the regulation and plan were never implemented, leaving an estimated 7% of Peurvian adults with dementia [15], without access to services. Five years later, a new law (30795) was enacted, repealing the previous one but lacking preventive measures. As of now, these plans signed by the government are still awaiting implementation.

The Peruvian case highlights the need for more than just an intention to improve population health through policies. There are additional challenges to overcome. Following successful examples of Costa Rica, Argentina and Chile, Peru could implement its own National Dementia Plan including prevention activities which are critical. Policy implementation is more likely close to the time of the policy launch when there is political and social focus. Unfortunately, in Peru, there seems to have been a loss of interest over time. Therefore, it is crucial to develop strategies to regain and sustain interest among elected officials and clinicians regarding aging and dementia in the country. Government stability and securing funding to support these policies are critical factors. The recent political instability in Peru, with 15 Ministers of Health holding office over the past 5 years, hinders policy progress. Broad community engagement is necessary from advocacy groups, clinicians, and caregivers of individuals with dementia to demonstrate the need for policy implementation. In Peru, high level policies will remain “stuck” unless there is a collective push from the bottom and from all sides. Broad community engagement is vital for improving the Peruvian National Plan for A/D and to assure the allocation of the necessary budget for prevention and research, ensuring its implementation, and monitoring and evaluating its impact.
